# Secondary Metabolites from the Soft Coral *Sinularia arborea*

**DOI:** 10.3390/md11093372

**Published:** 2013-09-03

**Authors:** Kuan-Hua Chen, Chang-Feng Dai, Mei-Chin Lu, Jan-Jung Li, Jih-Jung Chen, Yu-Chia Chang, Yin-Di Su, Wei-Hsien Wang, Ping-Jyun Sung

**Affiliations:** 1 Graduate Institute of Marine Biotechnology, National Dong Hwa University, Pingtung 944, Taiwan; E-Mails: asdfgh0213@gmail.com (K.-H.C.); jinx6609@nmmba.gov.tw (M.-C.L.); 2 National Museum of Marine Biology and Aquarium, Pingtung 944, Taiwan; E-Mails: jj@nmmba.gov.tw (J.-J.L.); jay0404@gmail.com (Y.-C.C.); gobetter04@yahoo.com.tw (Y.-D.S.); whw@nmmba.gov.tw (W.-H.W.); 3 Institute of Oceanography, National Taiwan University, Taipei 106, Taiwan; E-Mail: corallab@ntu.edu.tw; 4 Department of Pharmacy and Graduate Institute of Pharmaceutical Technology, Tajen University, Pingtung 907, Taiwan; E-Mail: jjchen@mail.tajen.edu.tw; 5 Doctoral Degree Program in Marine Biotechnology, National Sun Yat-sen University and Academia Sinica, Kaohsiung 804, Taiwan; 6 Department of Marine Biotechnology and Resources and Division of Marine Biotechnology, Asia-Pacific Ocean Research Center, National Sun Yat-sen University, Kaohsiung 804, Taiwan; 7 Natural Medicinal Products Research Center, China Medical University Hospital, Taichung 404, Taiwan; 8 Graduate Institute of Natural Products, Kaohsiung Medical University, Kaohsiung 807, Taiwan

**Keywords:** cembrane, *Sinularia arborea*, arbolide, crassarosterol, cytotoxicity

## Abstract

Two new 13-hydroxycembrane diterpenoids, arbolides A (**1**) and B (**2**), along with a known trihydroxysteroid, crassarosterol A (**3**), were isolated from the soft coral *Sinularia arborea*. The structures of new cembranes **1** and **2** were elucidated by spectroscopic methods. Steroid **3** was found to exhibit cytotoxicity toward K562 and MOLT-4 leukemia.

## 1. Introduction

**Figure 1:**
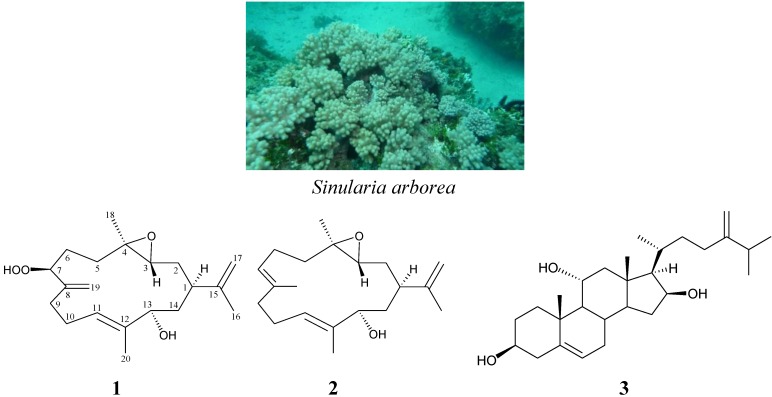
The soft coral *Sinularia arborea* and the structures of arbolides A (**1**), B (**2**) and crassarosterol A (**3**).

Previous studies on the chemical constituents of soft corals belonging to the genus *Sinularia* have led to the isolation of a number of interesting secondary metabolites and some of these were found to possess extensive bioactivities [1,2,3]. Continuation investigation on the chemical constituents of the marine invertebrates collected off the waters of Taiwan, two new cembrane-type diterpenoids, arbolides A (**1**) and B (**2**), and a known steroid, crassarosterol A (**3**) [4], were isolated from the soft coral *Sinularia arborea* (family Alcyonacea) (Figure 1). In this paper, we describe the isolation, structure determination and cytotoxicity of compounds **1**–**3**.

*Sinularia arborea*

## 2. Results and Discussion

Arbolide A (**1**) was isolated as a colorless oil that gave a pseudomolecular ion [M + Na]^+^ at *m/z* 359.2195 in the HRESIMS, indicating the molecular formula C_20_H_32_O_4_ (calcd for C_20_H_32_O_4_Na, 359.2198) (5° of unsaturation). The IR spectrum of **1** showed a broad band at 3345 cm^−1^, consistent with the presence of hydroxy group. The ^13^C NMR and DEPT spectra of **1** showed that this compound had 20 carbons (Table 1), including three methyls, six sp^3^ methylenes, two sp^2^ methylenes, four sp^3^ methines, an sp^2^ methine, an sp^3^ quaternary carbon and three sp^2^ quaternary carbons. The presence of a trisubstituted epoxide containing a methyl substituent was established from the NMR signals at δ_C_ 61.4 (CH), 61.1 (C) and δ_H_ 2.72 (1H, dd, *J* = 6.0, 6.0 Hz) and further confirmed by the proton signal of a methyl singlet at δ_H_ 1.27 (3H, s) (Table 1). A trisubstituted and two 1,1-disubstituted carbon-carbon double bonds were identified from the NMR signals at δ_C_ 138.4 (C), 125.3 (CH) and δ_H_ 5.48 (1H, br s); δ_C_ 147.3 (C), 113.2 (CH_2_) and δ_H_ 5.19 (1H, s), 5.15 (1H, s); δ_C_ 148.1 (C), 111.2 (CH_2_) and δ_H_ 4.75 (2H, br s), respectively. A hydroperoxy-bearing methine (δ_H_ 4.38, 1H, dd, *J* = 7.2, 4.8 Hz, δ_C_ 88.4, CH) [5,6,7] and a hydroxy-bearing methine (δ_H_ 3.92, 1H, dd, *J* = 6.0, 6.0 Hz, δ_C_ 76.4, CH) were identified from the characteristic NMR signal analysis. These data, combined with the five degrees of unsaturation implied by the molecular formula, suggested a bicyclic structure for **1**.

**Table 1:** ^1^H (400 MHz, CDCl_3_) and ^13^C (100 MHz, CDCl_3_) NMR data, ^1^H–^1^H COSY and HMBC correlations for cembrane **1**.

Position	δ_H_ (*J* in Hz)	δ_C_, Multiple	^1^H–^1^H COSY	HMBC
1	2.02 m	43.1, CH	H_2_-2, H_2_-14	C-2, -3, -13, -15, -17
2	1.89 m; 1.23 m	32.9, CH_2_	H-1, H-3	C-1, -3, -4, -14, -15
3	2.72 dd (6.0, 6.0)	61.4, CH	H_2_-2	C-2
4		61.1, C		
5	1.96 m; 1.30 m	33.7, CH_2_	H_2_-6	C-3, -4, -6, -7, -18
6	1.66 m; 1.52 m	28.6, CH_2_	H_2_-5, H-7	C-4, -5, -7, -8
7	4.38 dd (7.2, 4.8)	88.4, CH	H_2_-6	C-5, -6, -19
8		147.3, C		
9	2.24 m	31.3, CH_2_	H_2_-10, H_2_-19	C-8, -10, -11
10	2.41 m; 2.27 m	25.9, CH_2_	H_2_-9, H-11	C-9
11	5.48 br s	125.3, CH	H_2_-10, H_3_-20	n.o. *^a^*
12		138.4, C		
13	3.92 dd (6.0, 6.0)	76.4, CH	H_2_-14	C-1, -12, -14, -20
14	1.80 dd (6.8, 6.0)	38.2, CH_2_	H-1, H-13	C-1, -2, -12, -13, -15
15		148.1, C		
16	1.71 br s	18.8, CH_3_	H_2_-17	C-1, -15, -17
17	4.75 br s	111.2, CH_2_	H_3_-16	C-1, -15, -16
18	1.27 s	17.0, CH_3_		C-3, -4, -5
19	5.19 s; 5.15 s	113.2, CH_2_	H_2_-9	C-7, -8, -9
20	1.69 s	13.6, CH_3_	H-11	C-11, -12, -13
7-OOH	7.89 br s			n.o.

*^a^* n.o. = not observed.

**Figure 2:**
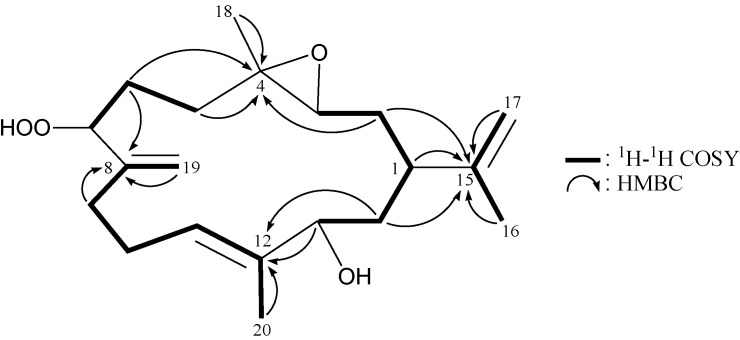
^1^H–^1^H COSY and selected HMBC correlations (protons→quaternary carbons) for **1**.

From the ^1^H–^1^H COSY spectrum of **1** (Table 1 and Figure 2), the separate spin systems of H-13/H_2_-14/H-1/H_2_-2/H-3, H_2_-5/H_2_-6/H-7 and H_2_-9/H_2_-10/H-11 were differentiated. These data, together with the key HMBC correlations between protons and quaternary carbons (Table 1 and Figure 2), such as H_2_-2, H_2_-5, H_2_-6, H_3_-18/C-4; H_2_-6, H_2_-9/C-8; H-13, H_2_-14/C-12; and H-1, H_2_-2, H_2_-14, H_2_-17/C-15, established the main carbon skeleton of **1**. The vinyl methyls at C-12 and C-15 were confirmed by the HMBC correlations between H_3_-20/C-11, -12, -13 and H_3_-16/C-1, -15, -17; and further supported by the allylic couplings between H-11/H_3_-20 and H_2_-17/H_3_-16 in the ^1^H–^1^H COSY spectrum of **1**. The exocyclic carbon-carbon double bonds at C-8 and C-15 were established by the HMBC correlations between H_2_-19/C-7, -8, -9 and H_2_-17/C-1, -15, -16; and further confirmed by the allylic couplings between H_2_-9/H_2_-19 and H_2_-17/H_3_-16 in the ^1^H–^1^H COSY spectrum of **1**. The hydroperoxy-bearing methine unit at δ_C_ 88.4 was more shielded than that expected for a hydroxy-bearing methine [5,6], and was correlated to the methine proton appearing at δ_H_ 4.38 in the HMQC spectrum. Thus, the remaining hydroxy group should be positioned at C-13, as indicated by the key ^1^H–^1^H COSY correlations and characteristic NMR signals. 

**Figure 3:**
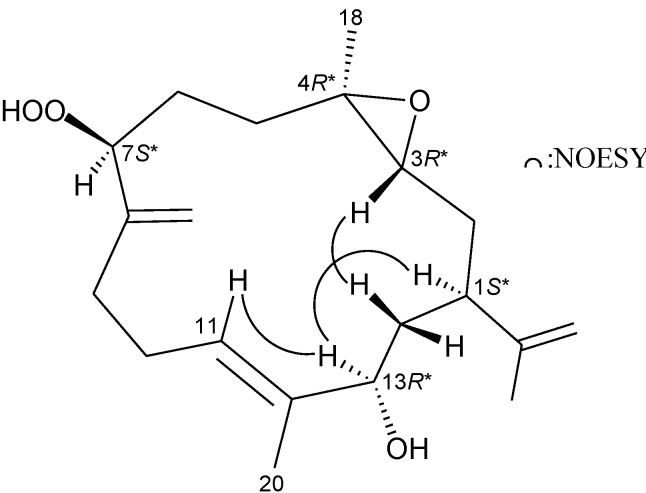
Key NOESY correlations of **1**.

The relative configuration of **1** was elucidated mainly from a NOESY spectrum. In the NOESY experiment for **1** (Figure 3), H-1 correlated with H-13, but not with H-3 and H_2_-14, and H-3 showed correlations with H_2_-14, revealing the S*-, R*- and R*-configurations of the chiral carbons C-1, C-3 and C-13, respectively, by modeling analysis. H-3 did not exhibit correlation with H_3_-18, reflecting the *trans* stereochemistry of 3,4-epoxide. Additionally, correlations between H-11 and H-13, as well as the lack of correlation between H-11/H_3_-20, reflected the *E* geometry of the double bond at C-11/12. Furthermore, by comparison of the proton chemical shift and coupling pattern of H-7 in **1** (δ_H_ 4.38 dd, *J* = 7.2, 4.8 Hz) with those of known cembrane analogues, manaarenolides A (δ_H_ 4.52, t, *J* = 3.5 Hz) and B (δ_H_ 4.40 dd, *J* = 11.5, 3.5 Hz), which were found to possess 7α- and 7β-hydroperoxy group in their structures, respectively [5], the 7-hydroperoxy group in **1** was proven to be β-oriented and possessing an S*-configuration. 

**Figure 4:**
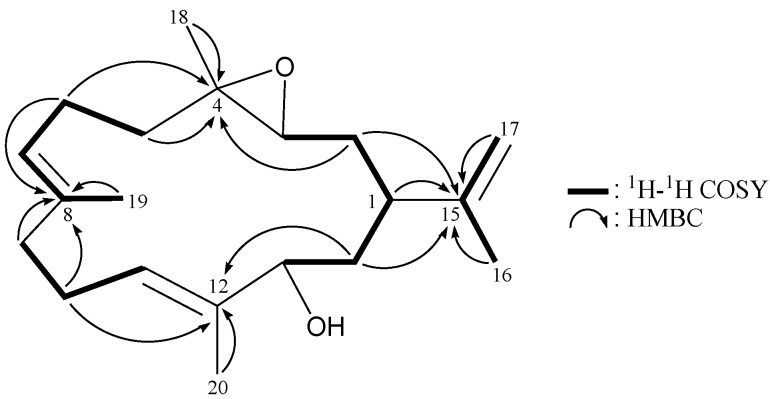
^1^H–^1^H COSY and selected HMBC correlations (protons→quaternary carbons) for **2**.

The HRESIMS spectrum of **2** (arbolide B) exhibited a pseudomolecular ion at *m/z* 327.2298 [M + Na]^+^, consistent with the molecular formula C_20_H_32_O_2_ and implying five degrees of unsaturation. The IR spectrum revealed the presence of hydroxy group (ν_max_3419 cm^−^^1^). The structure of cembrane **2** was deduced from its ^13^C NMR and DEPT spectra (Table 2), which showed that this compound has 20 carbons, including four methyls, seven methylenes (including an sp^2^ CH_2_), five methines (including two sp^2^ CH) and four quaternary carbons (including three sp^2^ quaternary carbons). From the ^1^H and ^13^C NMR spectra (Table 2), **2** was found to possess three olefinic groups (δ_H_ 5.05, 1H, ddq, *J* = 6.4, 6.4, 1.2 Hz; δ_C_ 123.2, CH; 135.1, C; δ_H_ 5.34, 1H, dd, *J* = 6.4, 6.4 Hz; δ_C_ 127.2, CH; 135.8, C; δ_H_ 4.71, 1H, dd, *J* = 2.0, 1.6 Hz; 4.64, 1H, dd, *J* = 1.6, 0.8 Hz; δ_C_ 147.6, C; 111.0, CH_2_). Signals at δ_C_ 63.3 (CH), 60.6 (C), 16.7 (CH_3_) and δ_H_ 2.74 (1H, dd, *J* = 10.0, 2.8 Hz), 1.23 (3H, s) revealed the presence of a methyl-containing trisubstituted epoxide. Detailed analysis of the ^1^H–^1^H COSY and HMBC correlations (Table 2 and Figure 4) further established the planar structure of **2** as a cembrane-type diterpenoid bearing a hydroxy group at C-13, two trisubstituted carbon-carbon double bonds at C-7/8 and C-11/12, a 1,1-disubstituted carbon-carbon double bond at C-15/17 and a methyl-containing epoxide at C-3/4.

**Table 2:** ^1^H (400 MHz, CDCl_3_) and ^13^C (100 MHz, CDCl_3_) NMR data, ^1^H–^1^H COSY and HMBC correlations for cembrane **2**.

Position	δ_H_ (*J* in Hz)	δ_C_, Multiple	^1^H–^1^H COSY	HMBC
1	2.01 m	40.7, CH	H_2_-2, H_2_-14	C-3, -13, -15
2	1.77 m; 1.31 m	33.5, CH_2_	H-1, H-3	C-1, -3, -4, -14, -15
3	2.74 dd (10.0, 2.8)	63.3, CH	H_2_-2	C-2
4		60.6, C		
5	2.06 m; 1.27 m	38.1, CH_2_	H_2_-6	C-3, -4, -6, -7
6	2.29 m; 2.10 m	24.0, CH_2_	H_2_-5, H-7	C-4, -5, -7, -8
7	5.05 ddq (6.4, 6.4, 1.2)	123.2, CH	H_2_-6, H_3_-19	C-6, -9, -19
8		135.1, C		
9	2.20 m; 2.04 m	38.8, CH_2_	H_2_-10	C-7, -8, -10, -11, -19
10	2.16 m	24.1, CH_2_	H_2_-9, H-11	C-8, -9, -11, -12
11	5.34 dd (6.4, 6.4)	127.2, CH	H_2_-10	C-9, -10, -13, -20
12		135.8, C		
13	3.87 br d (10.0)	75.3, CH	H_2_-14	C-11, -20
14	1.82 ddd (13.6, 10.8, 2.8) 1.73 ddd (13.6, 10.0, 3.6)	37.3, CH_2_	H-1, H-13	C-1, -2, -12, -15
15		147.6, C		
16	1.67 br s	18.5, CH_3_	H_2_-17	C-1, -15, -17
17	4.71 dd (2.0, 1.6); 4.64 dd (1.6, 0.8)	111.0, CH_2_	H_3_-16	C-1, -15, -16
18	1.23 s	16.7, CH_3_		C-3, -4, -5
19	1.63 br s	16.2, CH_3_	H-7	C-7, -8, -9
20	1.67 s	13.0, CH_3_		C-11, -12, -13

**Figure 5:**
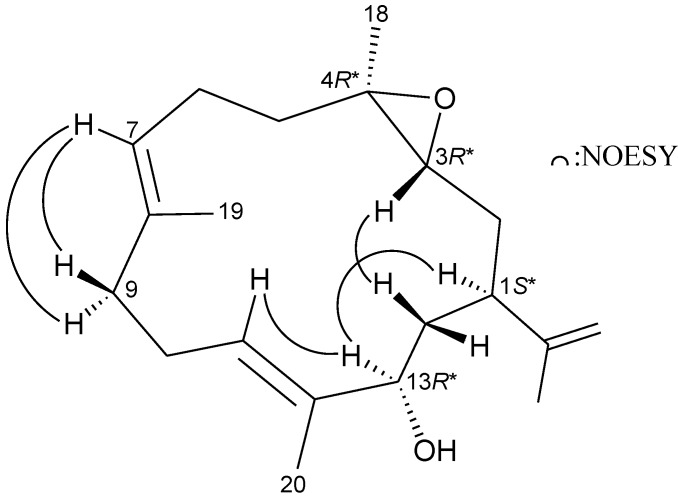
The NOESY correlations of **2**.

The relative structure of **2** was elucidated by analysis of NOESY correlations, as shown in Figure 5. In the NOESY experiment for **1**, H-1 correlated with H-13, but not with H-3, and H-3 showed correlations with H_2_-14, but not with H_3_-18 revealing the S*-, R*-, R*- and R*-configurations of the chiral carbons C-1, C-3, C-4 and C-13, respectively, by modeling analysis. Correlations observed between H-7/H_2_-9 and H-11/H-13, as well as the lack of correlation between H-7/H_3_-19 and H-11/H_3_-20, reflected the *E* geometry of the double bonds at C-7/8 and C-11/12.

The steroid **3** was found to be identical with the known compound, crassarosterol A, which was first isolated from a Formosan soft coral *Sinularia crassa*, on the basis of the comparison of its physical and spectroscopic data with those reported previously [4].

Cytotoxicity of compounds **1**–**3** toward K562 (human erythromyeloblastoid leukemia), MOLT-4 (human acute lymphoblastic leukemia), HTC-116 (human acute promyelocytic leukemia), DLD-1 (human colorectal adenocarcinoma), T-47D (human breast ductal carcinoma), MDA-MB-231 (human breast adenocarcinoma) and MCF-7 (human breast adenocarcinoma) cells was studied, and the results are shown in Table 3. These data showed that crassarosterol A (**3**) exhibited significant cytotoxicity towards K562 and MOLT-4 leukemia.

**Table 3:** Cytotoxic data of compounds **1**–**3**.

	Cell lines IC_50_ (μg/mL)						
Compounds	K562	MOLT-4	HTC-116	DLD-1	T-47D	MDA-MB-231	MCF-7
**1**	NA	NA	NA	NA	NA	NA	NA
**2**	NA	19.0	NA	NA	NA	NA	NA
**3**	2.5	0.7	19.0	NA	NA	NA	NA
**Doxorubicin** *^a^*	0.3	0.001	0.06	1.1	0.3	0.4	10.0

*^a^* Doxorubicin was used as a positive control. NA = not active at 20 μg/mL for 72 h.

## 3. Experimental Section

### 3.1. General Experimental Procedures

Optical rotations were measured at a Jasco P-1010 digital polarimeter (Japan Spectroscopic Corporation, Tokyo, Japan). Infrared spectra were recorded on a Varian Diglab FTS 1000 FT-IR spectrometer (Varian Inc. Palo Alto, CA, USA); peaks are reported in cm^−1^. NMR spectra were recorded on a Varian Mercury Plus 400 NMR spectrometer (Varian Inc.) using the residual CHCl_3_ signal (δ_H_ 7.26 ppm) as the internal standard for ^1^H NMR and CDCl_3_ (δ_C_ 77.1 ppm) for ^13^C NMR. Coupling constants (*J*) are given in Hz. ESIMS and HRESIMS were recorded using a Bruker APEX II mass spectrometer (Bruker, Bremen, Germany). Column chromatography was performed on silica gel (230–400 mesh, Merck, Darmstadt, Germany). TLC was carried out on precoated Kieselgel 60 F_254_ (0.25 mm, Merck); spots were visualized by spraying with 10% H_2_SO_4_ solution followed by heating. The normal phase HPLC (NP-HPLC) was performed using a system comprised of a Hitachi L-7110 pump (Hitachi Ltd. Tokyo, Japan) and a Rheodyne 7725 injection port (Rheodyne LLC, Rohnert Park, CA, USA). Two normal phase columns (Supelco Ascentis^®^ Si Cat #:581515-U, 25 cm × 21.2 mm, 5 μm; 581514-U, 25 cm × 10 mm, 5 μm, Sigma-Aldrich. Com. St. Louis, MO, USA) were used for NP-HPLC.

### 3.2. Animal Material

Specimens of the octocoral *Sinularia arborea* [8] were collected by hand using scuba equipment off the coast of southern Taiwan in October, 2012, and stored in a freezer (−20 °C) until extraction. A voucher specimen (NMMBA-TWSC-1200X) was deposited in the National Museum of Marine Biology and Aquarium, Taiwan.

### 3.3. Extraction and Isolation

Specimens of the soft coral *Sinularia arborea* (wet weight 1.6 kg, dry weight 576 g) were minced and extracted with ethyl acetate (EtOAc). The EtOAc extract left after removal of the solvent (12.5 g) was separated by silica gel and eluted using a mixture of *n*-hexane/EtOAc in a stepwise fashion from 100:1–pure EtOAc to yield 11 fractions A–K. Fraction G was separated by NP-HPLC, using a mixture of *n*-hexane and acetone (6:1) to yield 26 subfractions G1–G26. Fraction G13 was repurified by NP-HPLC, using a mixture of *n*-hexane and EtOAc (4:1) to yield 9 subfractions G13A–G13I. Fraction G13G was repurified by NP-HPLC, using a mixture of *n*-hexane and acetone (7:2, flow rate: 2.0 mL/min) to yield **2** (3.7 mg, *t*_R_ = 8 m). Fraction G13I was repurified by NP-HPLC, using a mixture of dichloromethane and acetone (10:1, flow rate: 1.0 mL/min) to yield **1** (2.9 mg, *t*_R_ = 332 m). Fraction H was purified by NP-HPLC, using a mixture of *n*-hexane and EtOAc (2:1) to obtain 14 subfractions H1–H14. Fraction H12 was repurified by NPLC, using a mixture of *n*-hexane and acetone (3:1, flow rate: 2.0 mL/min) to yield **3** (1.2 mg, *t*_R_ = 149 m). 

Arbolide A (**1**): colorless oil; 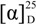 +12 (*c* 0.15, CHCl_3_); IR (neat) ν_max_ 3445 cm^−1^; ^1^H (400 MHz, CDCl_3_) and ^13^C (100 MHz, CDCl_3_) NMR data, see Table 1; ESIMS: *m/z* 359 [M + Na]^+^; HRESIMS: *m/z* 359.2195 (calcd for C_20_H_32_O_4_Na, 359.2198).

Arbolide B (**2**): colorless oil; 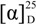 −3 (*c* 0.19, CHCl_3_); IR (neat) ν_max_ 3419 cm^−1^; ^1^H (400 MHz, CDCl_3_) and ^13^C (100 MHz, CDCl_3_) NMR data, see Table 2; ESIMS: *m/z* 327 [M + Na]^+^; HRESIMS: *m/z* 327.2298 (calcd for C_20_H_32_O_2_Na, 327.2300).

Crassarosterol A (**3**): white powder; 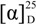 −16 (*c* 0.06, CHCl_3_) (ref. [4], 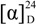 −45 (*c* 0.66, CHCl_3_)); IR (neat) ν_max_ 3396 cm^−1^; ESIMS: *m/z* 453 [M + Na]^+^. The ^1^H and ^13^C NMR data of **3** are in full agreement with those reported previously [4]. 

### 3.4. Cytotoxicity Testing

Cytotoxicity of compounds **1**–**3** was assayed with a modification of the MTT [3-(4,5-dimethylthiazol-2-yl)-2,5-diphenyltetrazolium bromide] colorimetric method. Cytotoxicity assays were carried out according to previously described procedures [9,10].

## 4. Conclusions

In our continuing investigation on the chemical constituents of marine invertebrates collected off the waters of Taiwan, the soft coral *Sinularia arborea* has resulted in the isolation of two new cembrane-type diterpenoids, arbolides A (**1**) and B (**2**) and a known trihydroxysteroid crassarosterol A (**3**) [4]. Steroid **3** was found to exhibit selective cytotoxicity toward the human leukemia K562 and MOLT-4 cells. To the best of our knowledge, this is the first time that the natural substances from *Sinularia arborea* have been reported.

## Supplementary Files

Supplementary File 1 Supplementary Materials (PDF, 897 KB)
