# Supplementary material for: Secondary Metabolites from the Soft Coral Sinularia arborea
Source: Mar Drugs. 2013 Sep 3;11(9):3372–80. doi: 10.3390/md11093372 (PMC3801123; doi:10.3390/md11093372)

Supplementary Materials

Figure S1. ^{1}H NMR spectrum (400 MHz) of compound **1** in $CDCl_3$. 2
Figure S2. ^{13}C NMR spectrum (100 MHz) of compound **1** in $CDCl_3$. 2
Figure S3. HSQC spectrum (400 MHz) of compound **1** in $CDCl_3$. 3
Figure S4. HMBC spectrum (400 MHz) of compound **1** in $CDCl_3$. 3
Figure S5. COSY spectrum (400 MHz) of compound **1** in $CDCl_3$. 4
Figure S6. NOESY spectrum (400 MHz) of compound **1** in $CDCl_3$. 4
Figure S7. ^{1}H NMR spectrum (400 MHz) of compound **2** in $CDCl_3$. 5
Figure S8. ^{13}C NMR spectrum (100 MHz) of compound **2** in $CDCl_3$. 5
Figure S9. HSQC spectrum (400 MHz) of compound **2** in $CDCl_3$. 6
Figure S10. HMBC spectrum (400 MHz) of compound **2** in $CDCl_3$. 6
Figure S11. COSY spectrum (400 MHz) of compound **2** in $CDCl_3$. 7
Figure S12. NOESY spectrum (400 MHz) of compound **2** in $CDCl_3$. 7
Figure S13. ^{1}H NMR spectrum (400 MHz) of compound **3** in $CDCl_3$. 8
Figure S14. ^{13}C NMR spectrum (100 MHz) of compound **3** in $CDCl_3$. 8
Figure S15. HSQC spectrum (400 MHz) of compound **3** in $CDCl_3$. 9
Figure S16. HMBC spectrum (400 MHz) of compound **3** in $CDCl_3$. 9
Figure S17. COSY spectrum (400 MHz) of compound **3** in $CDCl_3$. 10
Figure S18. NOESY spectrum (400 MHz) of compound **3** in $CDCl_3$. 10

Figure S1. ^{1}H NMR spectrum (400 MHz) of compound **1** in $CDCl_3$.

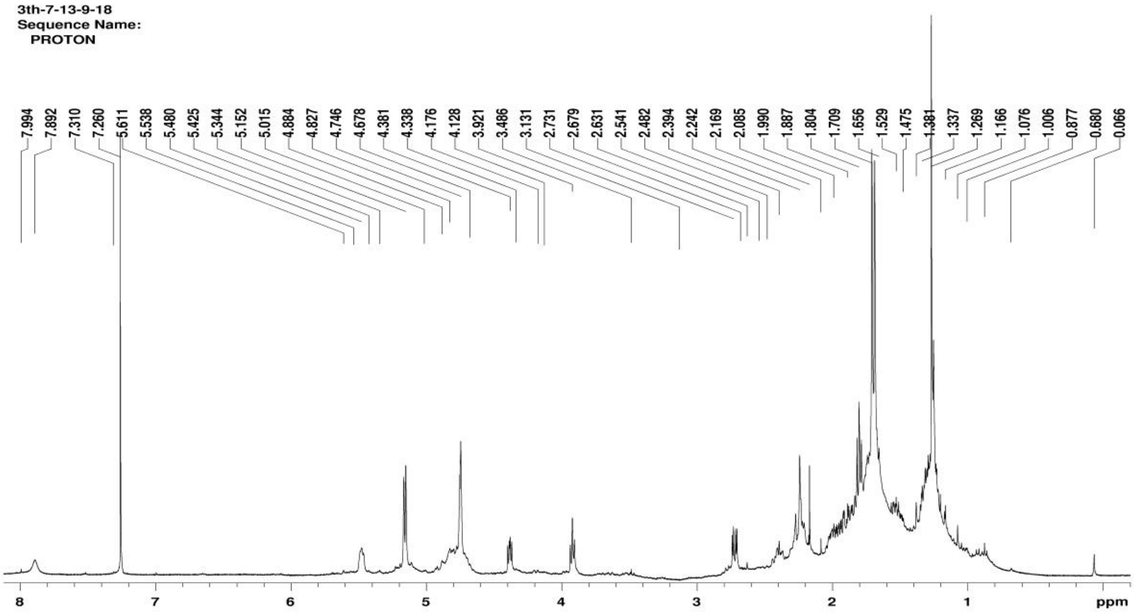

Figure S2. ^{13}C NMR spectrum (100 MHz) of compound **1** in $CDCl_3$.

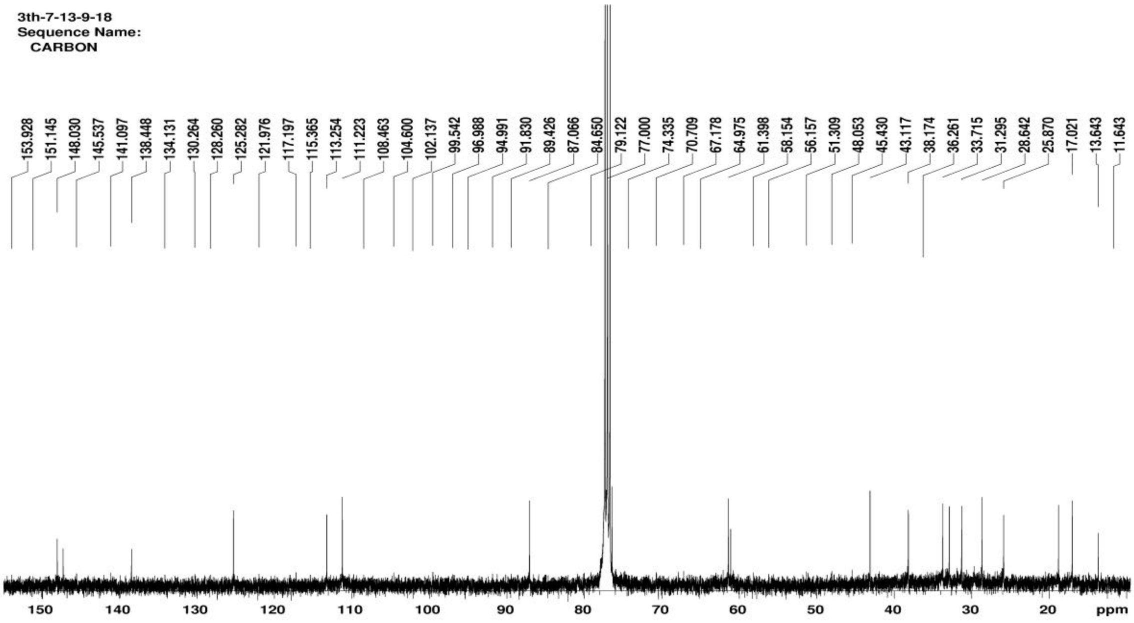

Figure S3. HSQC spectrum (400 MHz) of compound **1** in $CDCl_3$.

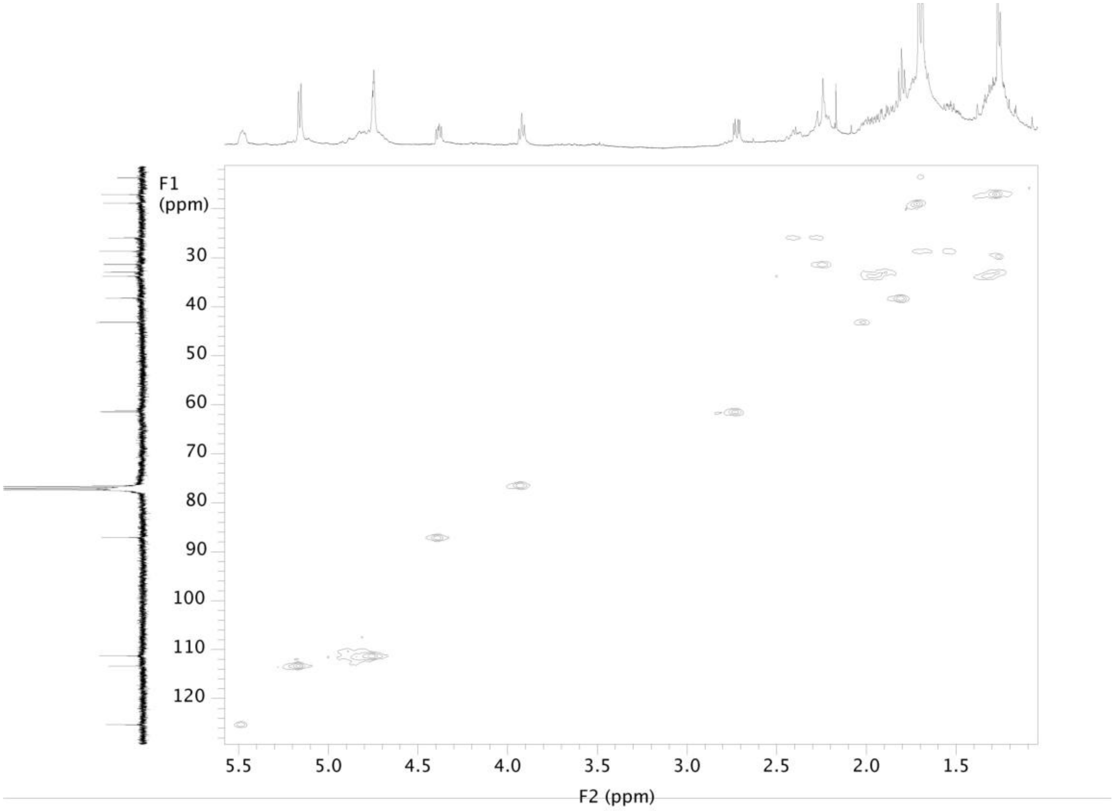

Figure S4. HMBC spectrum (400 MHz) of compound **1** in $CDCl_3$.

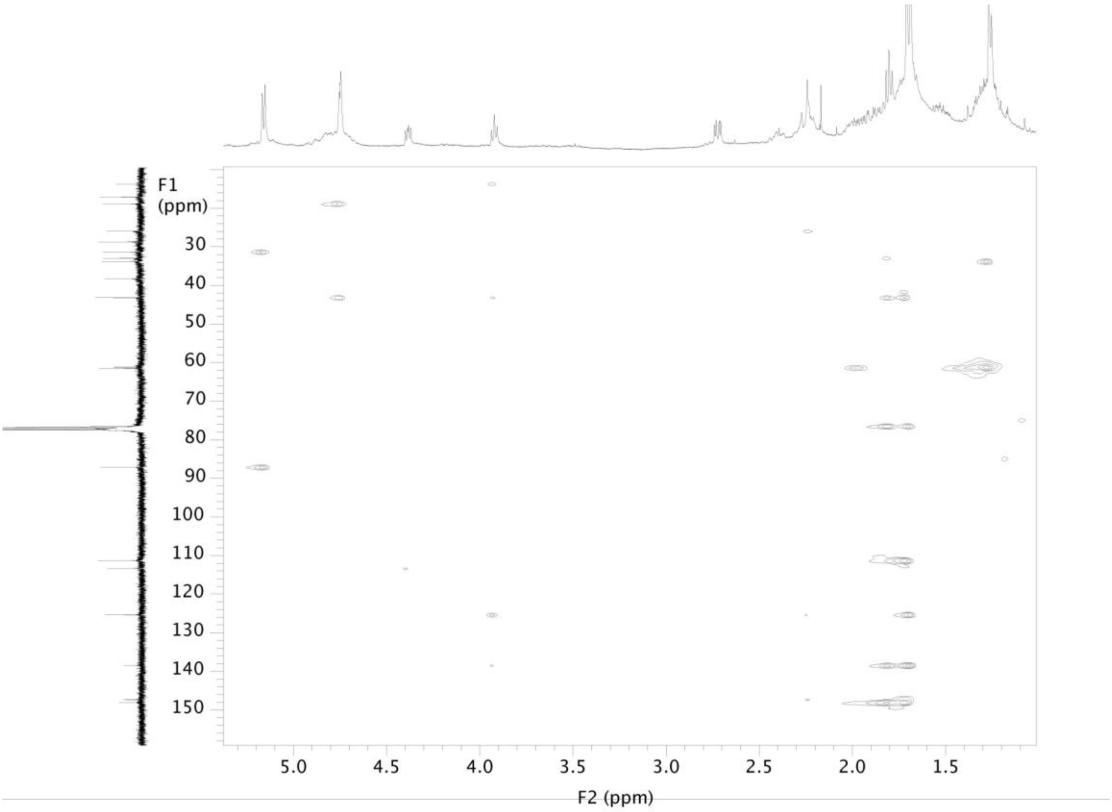

Figure S5. COSY spectrum (400 MHz) of compound **1** in $CDCl_3$.

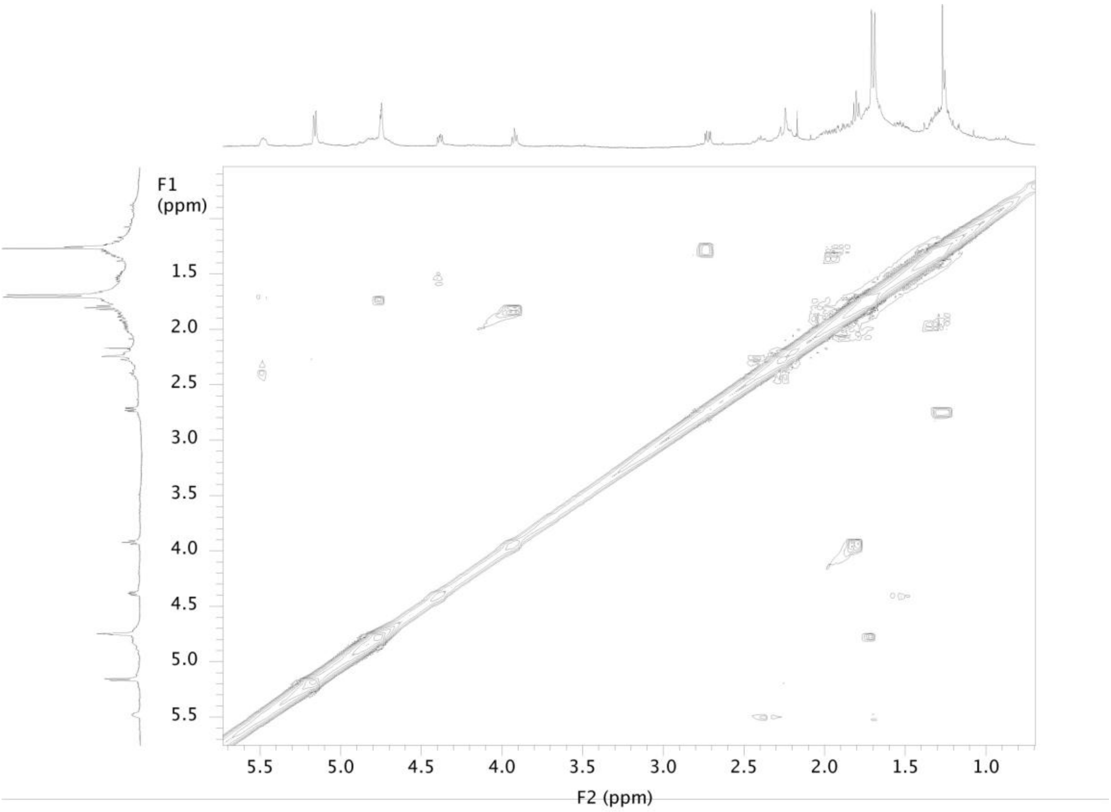

Figure S6. NOESY spectrum (400 MHz) of compound **1** in $CDCl_3$.

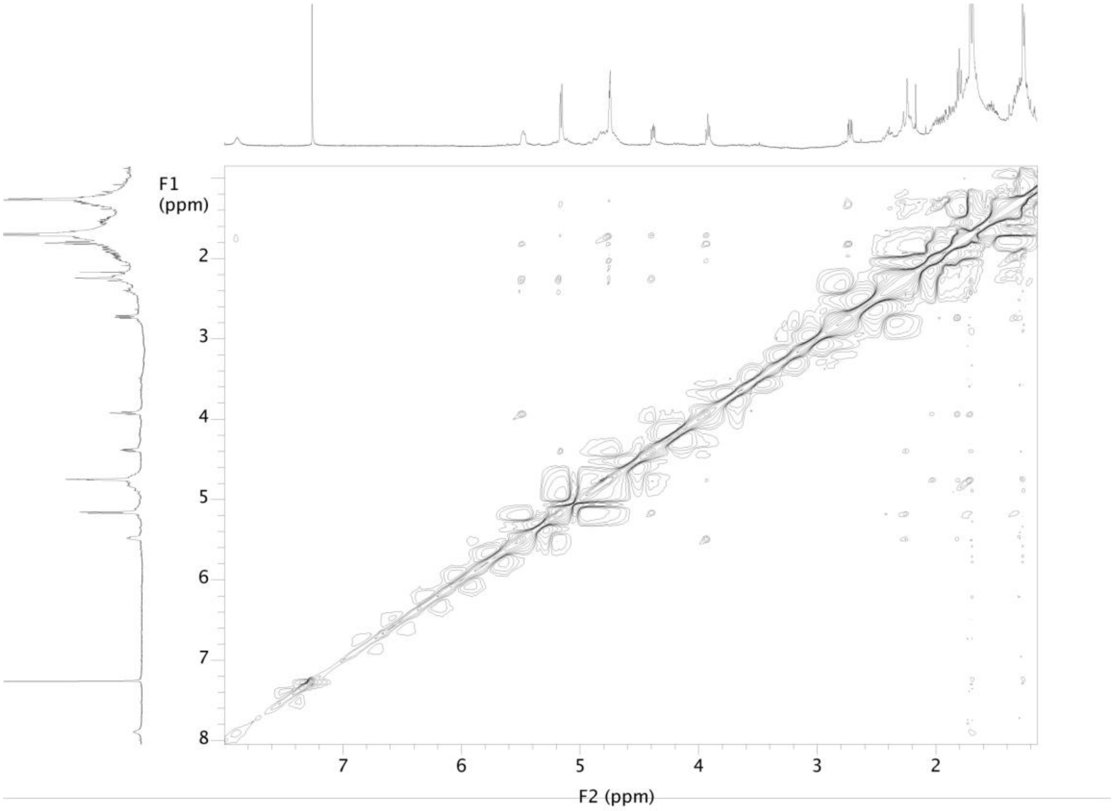

Figure S7. ^{1}H NMR spectrum (400 MHz) of compound **2** in $CDCl_3$.

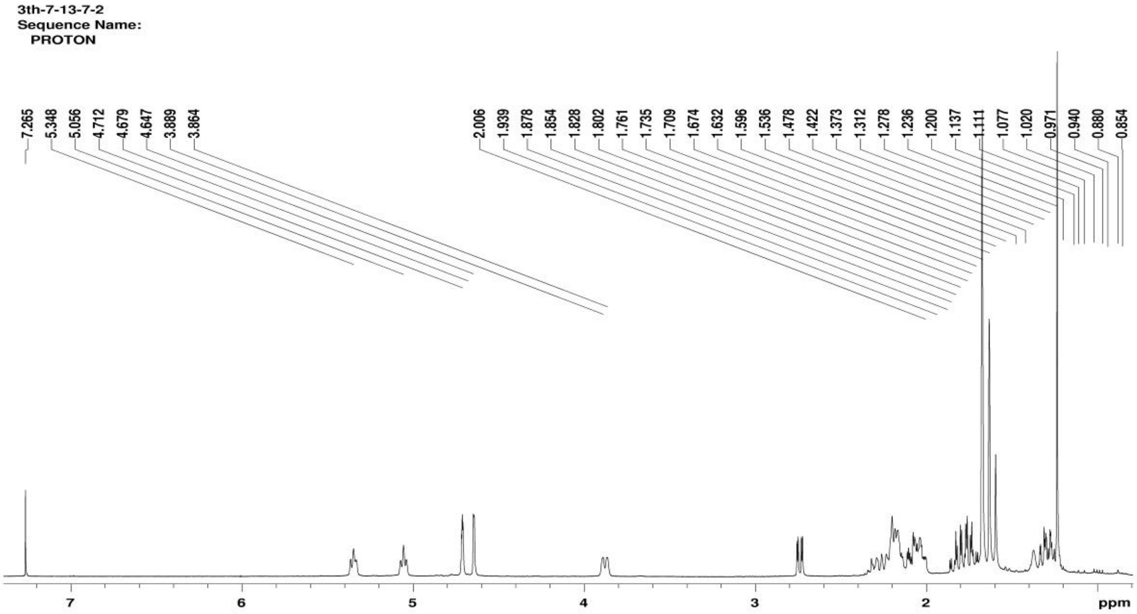

Figure S8. ^{13}C NMR spectrum (100 MHz) of compound 2 in $CDCl_3$.

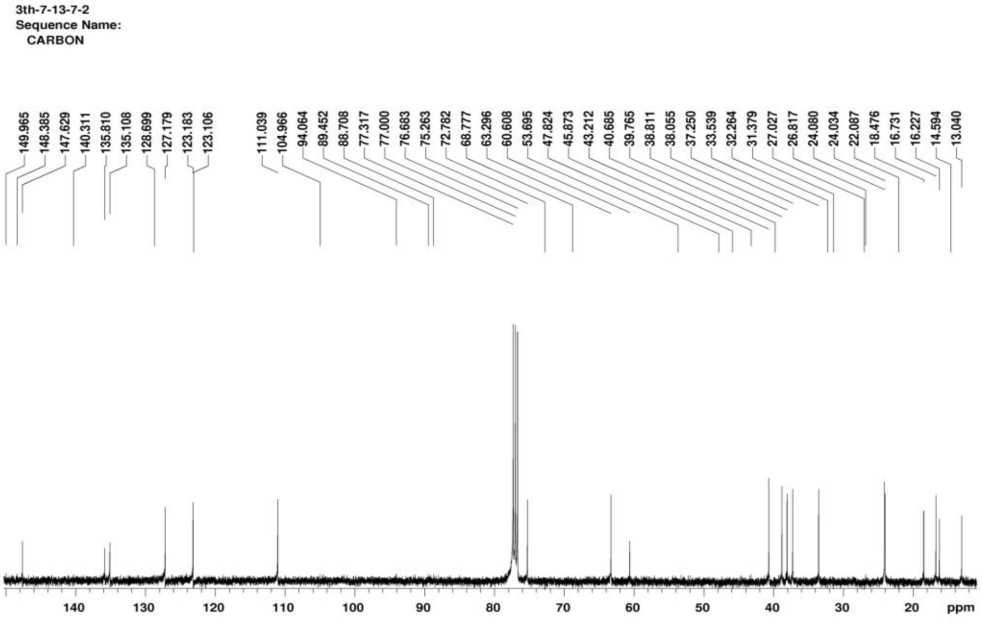

Figure S9. HSQC spectrum (400 MHz) of compound **2** in $CDCl_3$.

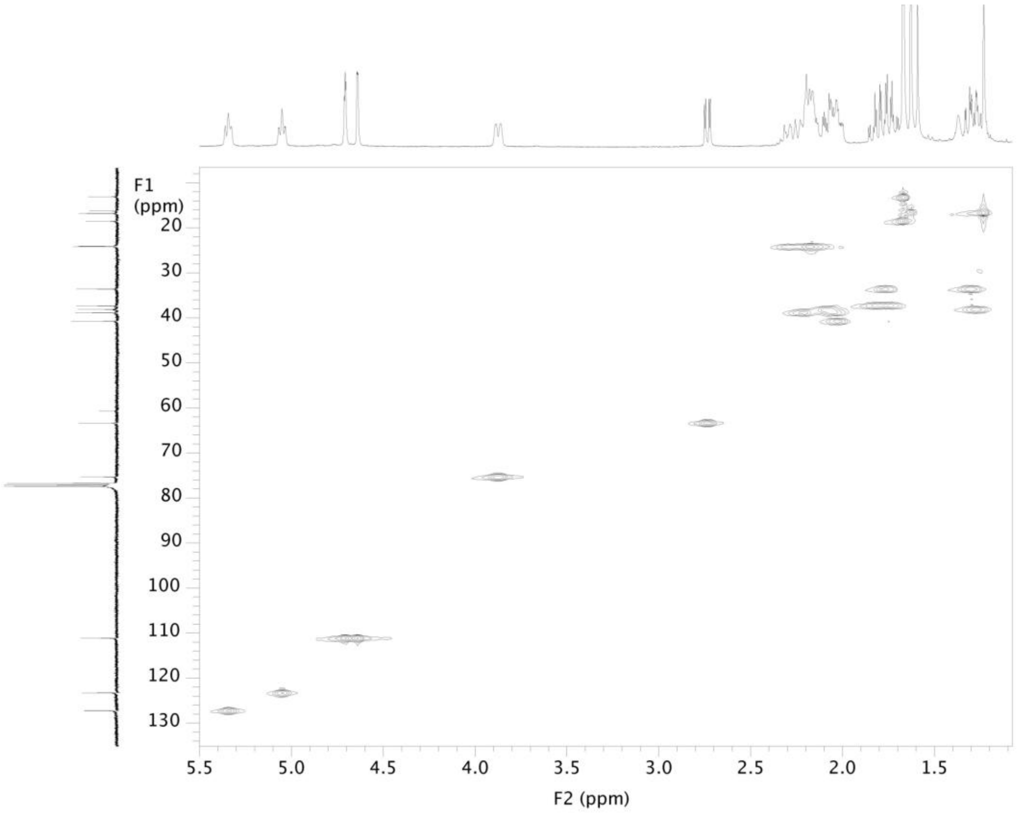

Figure S10. HMBC spectrum (400 MHz) of compound **2** in $CDCl_3$.

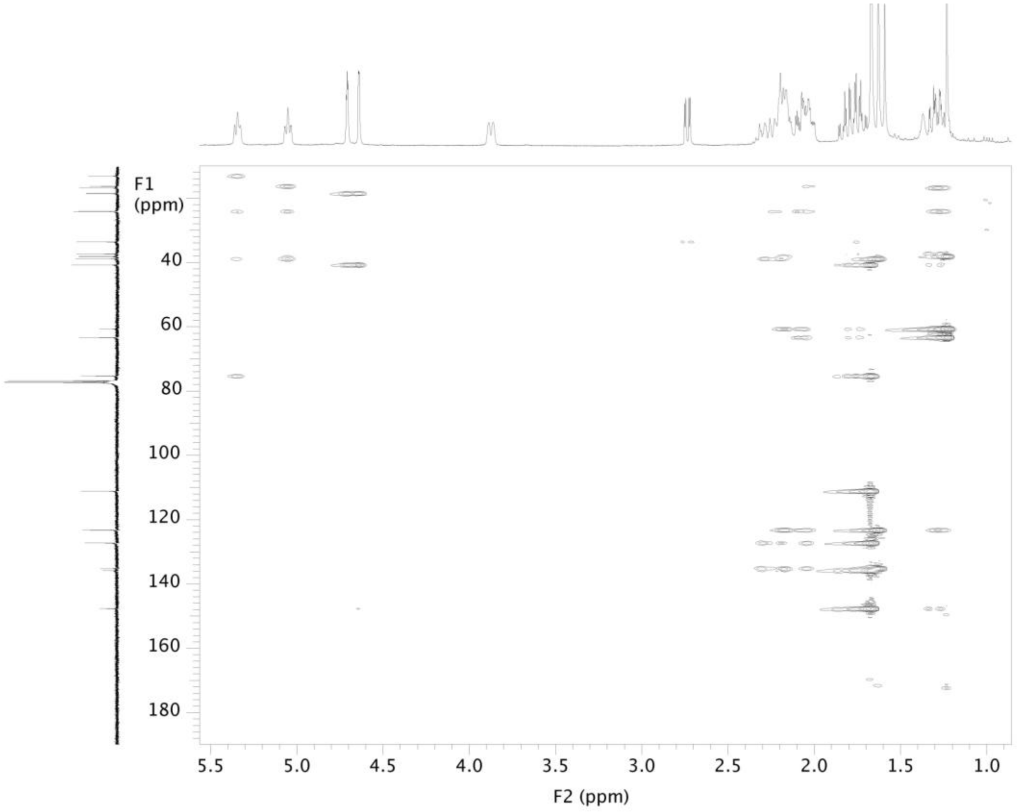

Figure S11. COSY spectrum (400 MHz) of compound **2** in $CDCl_3$.

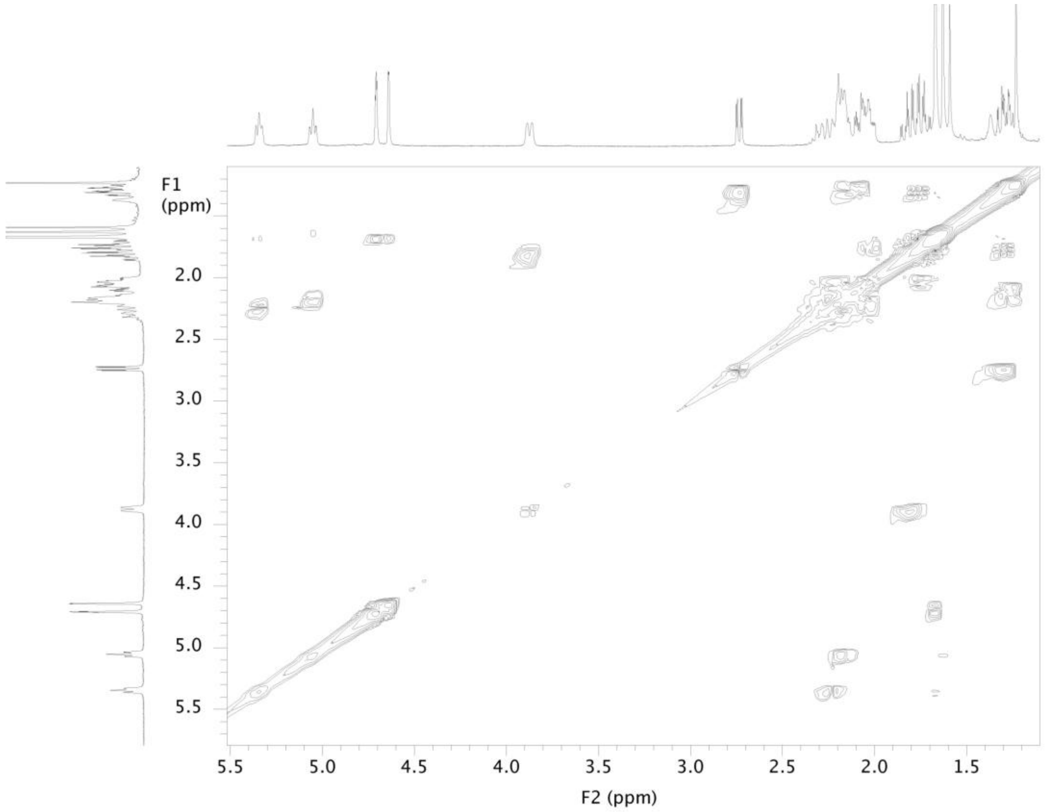

Figure S12. NOESY spectrum (400 MHz) of compound **2** in CDCl3.

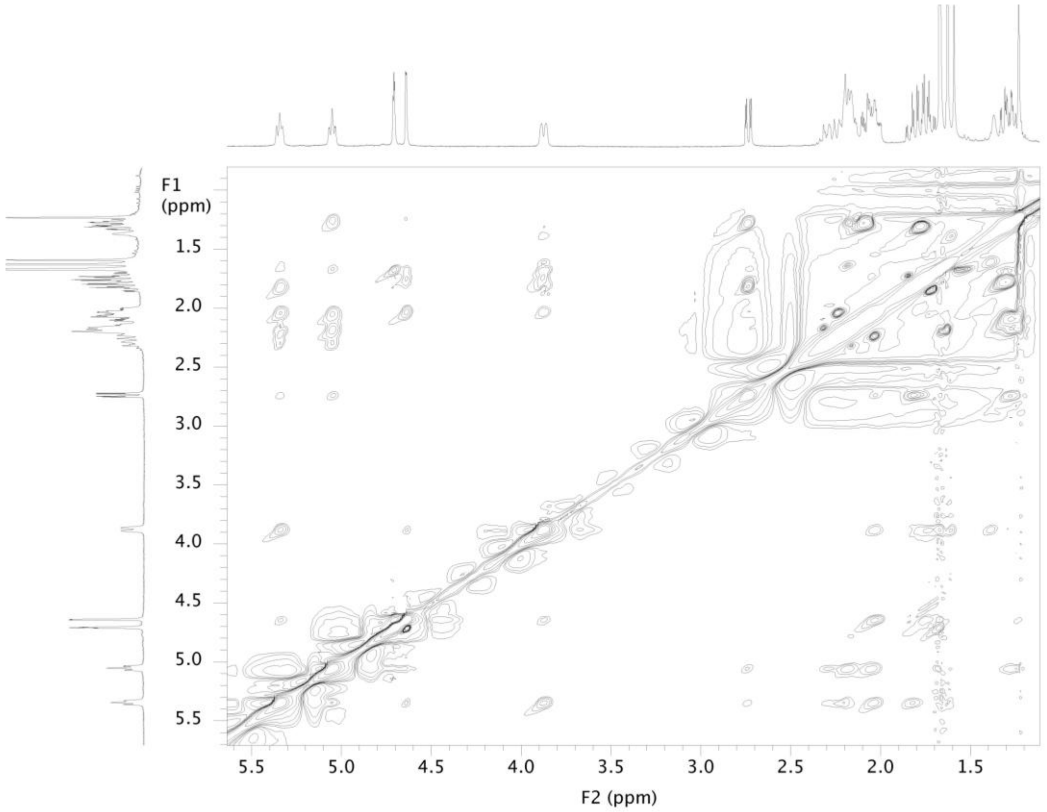

Figure S13. ^{1}H NMR spectrum (400 MHz) of compound **3** in $CDCl_3$.

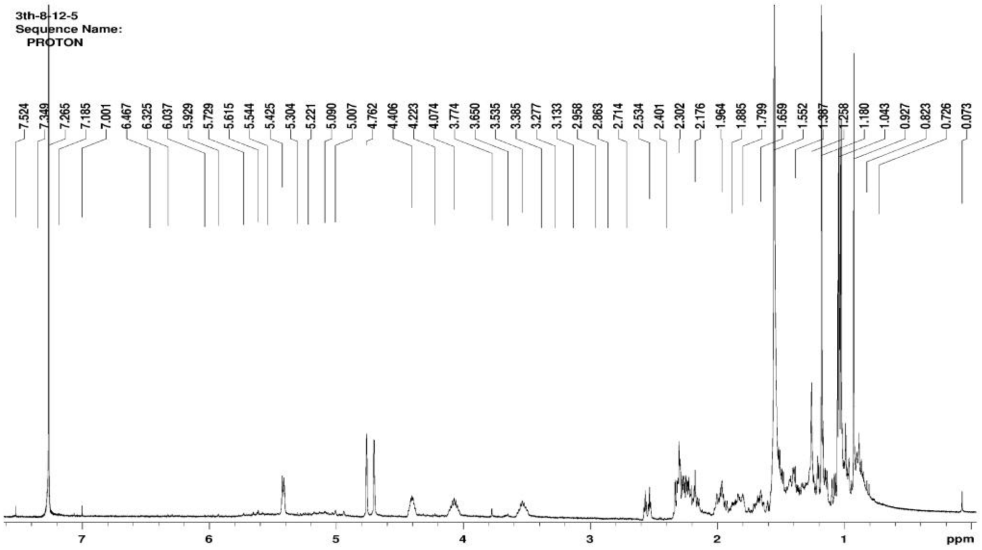

Figure S14. ^{13}C NMR spectrum (100 MHz) of compound **3** in $CDCl_3$.

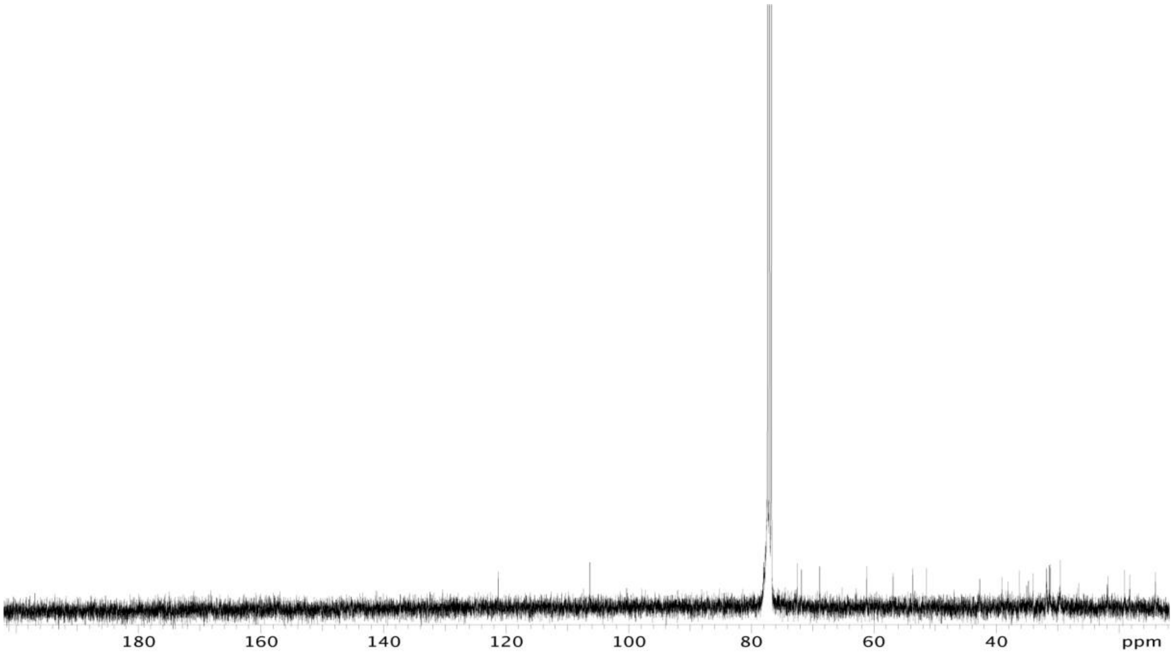

Figure S15. HSQC spectrum (400 MHz) of compound **3** in $CDCl_3$.

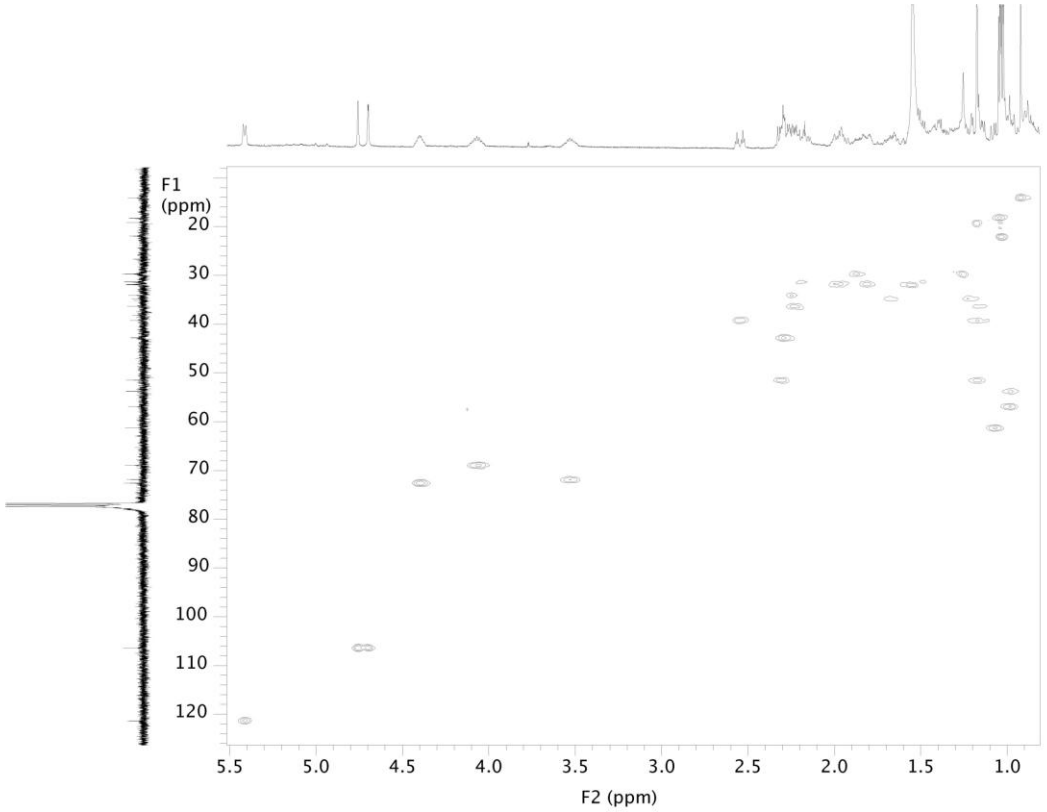

Figure S16. HMBC spectrum (400 MHz) of compound **3** in $CDCl_3$.

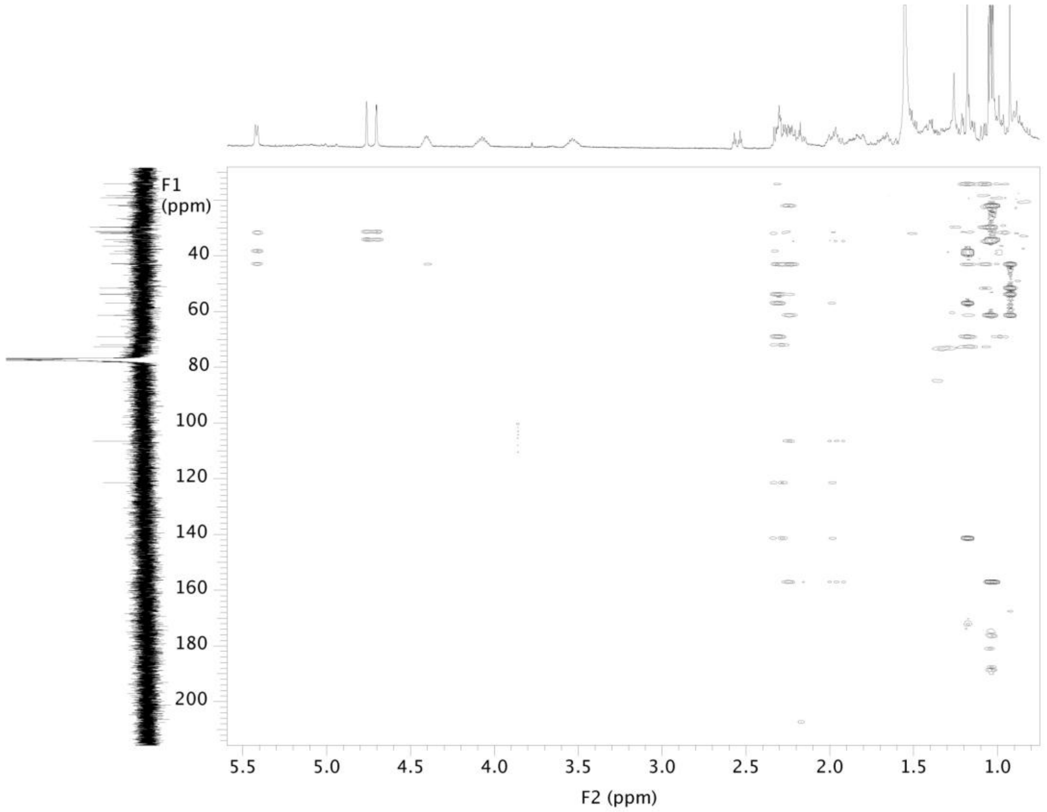

Figure S17. COSY spectrum (400 MHz) of compound **3** in $CDCl_3$.

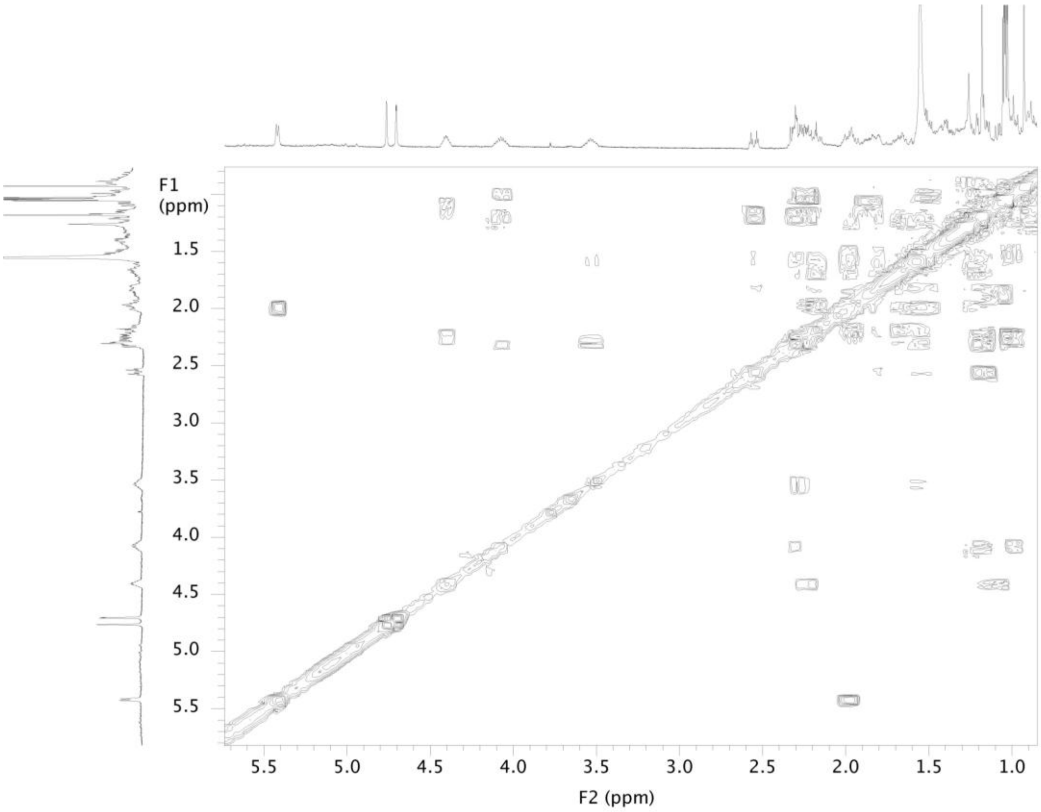

Figure S18. NOESY spectrum (400 MHz) of compound **3** in $CDCl_3$.

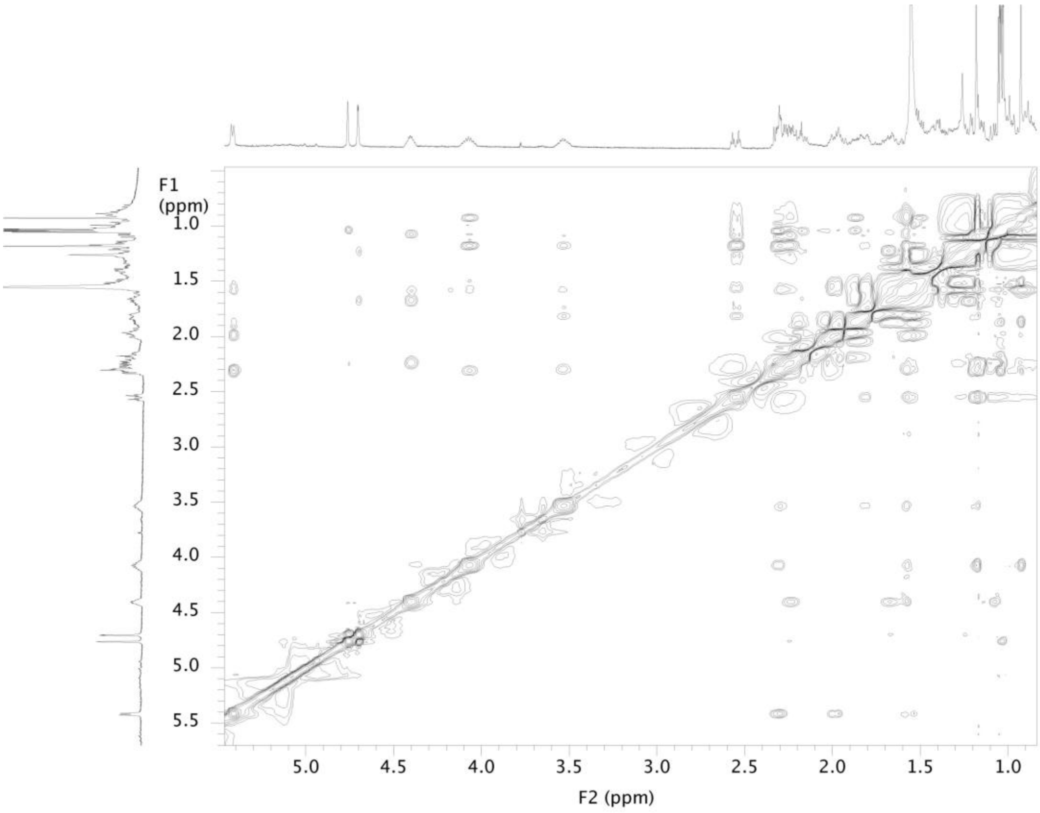

Supplement: Supplementary File 1 — Supplementary Materials (PDF, 897 KB) [file marinedrugs-11-03372-s001.pdf]
